# CSGM Designer: a platform for designing cross-species intron-spanning genic markers linked with genome information of legumes

**DOI:** 10.1186/s13007-015-0074-6

**Published:** 2015-04-18

**Authors:** Jin-Hyun Kim, Chaeyoung Lee, Daejin Hyung, Ye-Jin Jo, Joo-Seok Park, Douglas R Cook, Hong-Kyu Choi

**Affiliations:** Department of Medical Bioscience, Dong-A University, Saha-Gu Nakdong-Daero 550 beongil 37, Busan, 604-714 Republic of Korea; Department of Computer Science, Dong-A University, Saha-Gu Nakdong-Daero 550 beongil 37, Busan, 604-714 Republic of Korea; Department of Genetic Engineering, Dong-A University, Saha-Gu Nakdong-Daero 550 beongil 37, Busan, 604-714 Republic of Korea; Department of Applied Bioscience, Dong-A University, Saha-Gu Nakdong-Daero 550 beongil 37, Busan, 604-714 Republic of Korea; Department of Plant Pathology, University of California, One Shields Ave, Davis, CA 95616 USA

**Keywords:** Marker design, Genic marker, Legumes, eHT-PCR, Circular viewer

## Abstract

**Background:**

Genetic markers are tools that can facilitate molecular breeding, even in species lacking genomic resources. An important class of genetic markers is those based on orthologous genes, because they can guide hypotheses about conserved gene function, a situation that is well documented for a number of agronomic traits. For under-studied species a key bottleneck in gene-based marker development is the need to develop molecular tools (e.g., oligonucleotide primers) that reliably access genes with orthology to the genomes of well-characterized reference species.

**Results:**

Here we report an efficient platform for the design of cross-species gene-derived markers in legumes. The automated platform, named CSGM Designer (URL: http://tgil.donga.ac.kr/CSGMdesigner), facilitates rapid and systematic design of cross-species genic markers. The underlying database is composed of genome data from five legume species whose genomes are substantially characterized. Use of CSGM is enhanced by graphical displays of query results, which we describe as “circular viewer” and “search-within-results” functions. CSGM provides a virtual PCR representation (eHT-PCR) that predicts the specificity of each primer pair simultaneously in multiple genomes. CSGM Designer output was experimentally validated for the amplification of orthologous genes using 16 genotypes representing 12 crop and model legume species, distributed among the galegoid and phaseoloid clades. Successful cross-species amplification was obtained for 85.3% of PCR primer combinations.

**Conclusion:**

CSGM Designer spans the divide between well-characterized crop and model legume species and their less well-characterized relatives. The outcome is PCR primers that target highly conserved genes for polymorphism discovery, enabling functional inferences and ultimately facilitating trait-associated molecular breeding.

**Electronic supplementary material:**

The online version of this article (doi:10.1186/s13007-015-0074-6) contains supplementary material, which is available to authorized users.

## Background

Genetic markers and mapping are a principal means to study the genetic features of organisms. In the case of crop species, genetic markers can facilitate the introgression of desirable traits among accessions and species, and they provide the basis of marker-assisted trait dissection (e.g., QTL analysis). Genetic markers were first employed in 1932 based on isozyme analysis and progressed to DNA based markers as electrophoretic methods became practical in the early 1980’s [1]. In the absence of DNA sequence data, anonymous genetic markers (e.g., restriction fragment length polymorphisms [RFLP] and related methods) were the dominant technologies [2]. With the advent of genome sequencing projects, it became feasible to develop genetic markers that amplified common genes among related species. Initially such approaches depended on transcript sequence (e.g., Expressed Sequence Tags), which in many cases is now supplanted by the availability of whole genome sequence (WGS) information. For sequence-characterized genomes, high throughput genotyping-by-sequencing (GBS) methods eliminate the need to create individual genetic markers. However, numerous species lack sufficient genome data for GBS methods, and in these cases the use of PCR amplification remains an important tool for marker development. Genetic markers that target orthologous genes are particularly useful, because they permit cross-species inferences (e.g., of structural and conservation), and this approach has been used widely in legumes [3] and other species.

The enormity of genomic information currently available in public databases is both an asset and a deterrent to large-scale molecular marker development. On the one hand such information provides the basis of systematic identification of orthologous genes and corresponding tools (e.g., PCR primers), while on the other hand doing so requires organized bioinformatics activities not available for many small research groups working on under-characterized species. In the absence of such bioinformatic activities, development of genetic markers remains laborious and time-consuming. Of particular interest are cross-species gene-targeted markers, which have utility in translating marker information across different crop plants, however such markers can be technically difficult to design.

Towards this end, we focus on the Fabaceae. The Fabaceae (legume family) is the third largest family among flowering plants and the second most important group of crop plants, second only to the Poaceae (grass family). Legumes consist of approximately 20,000 species among three subfamilies, the Caesalpionoideae, Mimosoideae and Papilionoideae [4]. Within the most recently diverged Papilionoideae subfamily, the galegoid and phaseoloid/ millettioid clades contain the vast majority of agricultural species. Several model and crop legumes derive from these two clades and many of these species have substantially complete genome sequences, including *Medicago truncatula* [5], *Lotus japonicus* [6], *Glycine max* [7], *Phaseolus vulgaris* [8] and *Cajanus cajan* [9]. Because the phylogenetic space encompassed by these species includes the majority of crop legumes, their genome data provides an opportunity to identify large sets of genes that are also common to related uncharacterized legume species, and on this basis one can develop genetic markers that are operative for a range of legume crops that lack genomic resources.

Here we describe the “CSGM (cross-species genic marker) Designer” web resource, a legume-focused platform for the systematic design of cross-species genic markers. To enhance ease of use we employed graphic visualization tools. The “circular viewer” pane depicts the attributes of numerous competing PCR primer candidates to facilitate selection by the user. The eHT (electronic high throughput)-PCR pane reports the results of *in silico* PCR at the genome-wide scale, which aids the selection of primer pairs most likely to yield specific-versus-nonspecific PCR products prior to laboratory validation.

## Results

### Conceptual implementation of cross-species intron-spanning genic marker designing platform

**Figure 1:**
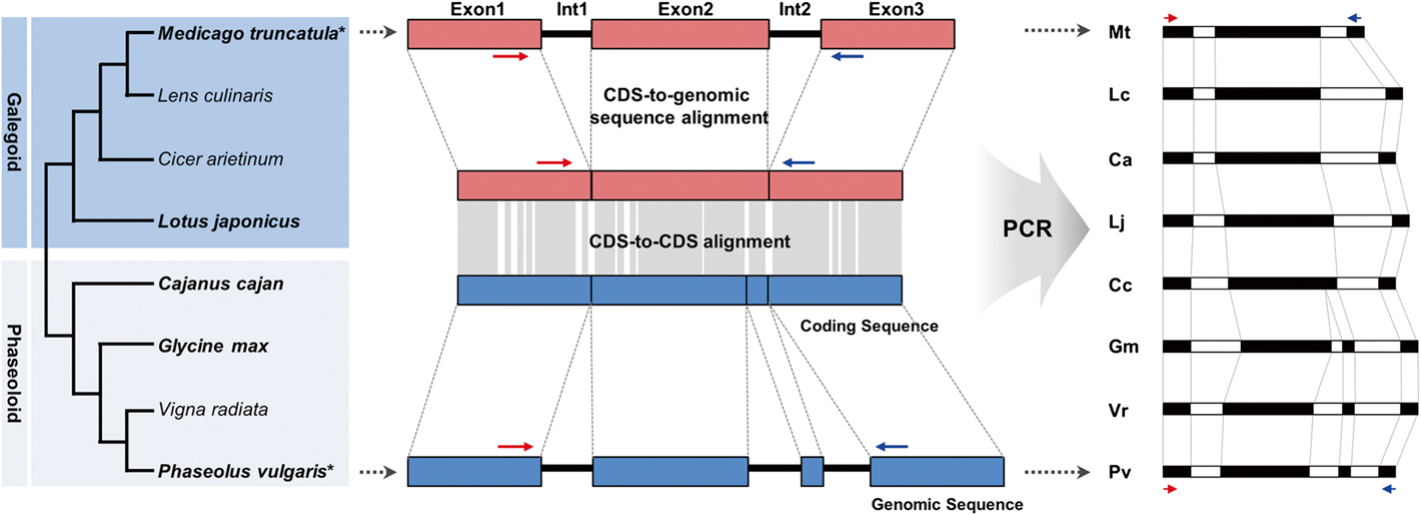
Conceptual design of cross-species intron-spanning genic markers. Five fully sequenced legume species used to construct the database are denoted in bold italic. Two species, *M. truncatula* and *P. vulgaris*, which were used to anchor the two clades, are highlighted with the asterisk superscript. Conserved regions in the CDS-to-CDS alignment, where are the sites for cross-amplifiable PCR primer pairs, are depicted using grey boxes.

CSGM Designer implements an automated platform to design gene-targeted molecular markers, with the goal of creating PCR amplicons that operate across multiple evolutionarily diverged, but related species (Figure 1). Towards this end, CSGM Designer focuses on PCR primer design within exon regions, where nucleotide sequence divergence is typically more constrained than non-protein coding regions. To accommodate the need for high rates of polymorphism within crop germplasm, which is of low genetic diversity, CSGM Designer identifies primer pairs that amplify across less constrained intron sequences, thus enhancing the success rate of marker development. The position of exon-intron junctions can be accurately detected by aligning genomic sequence with the corresponding CDS (Figure 1), permitting CSGM Designer to avoid primer design over splice junctions that would preclude PCR amplification. The best-conserved exon regions between two aligned orthologous genes are analyzed to identify DNA sequence regions with the lowest level of mismatch, and these highly conserved intervals become the template for primer design.

Maintaining specificity in gene-targeted PCR amplification is frequently hampered by the presence of numerous close paralogs within the target genome, a particular problem for large gene families. PCR products that include multi-genomic loci are not readily distinguished by electrophoretic separation, unless primer pairs are selected to generate paralog-specific amplicon size variation. Thus CSGM Designer includes a PCR simulation module to predict PCR amplicon size with given primer pairs. In addition, the platform is delivered through an interactive web-based GUI structured to guide users to efficient selection of PCR primer pairs.

### Construction of legume genomic information-linked genic marker design platform

**Figure 2:**
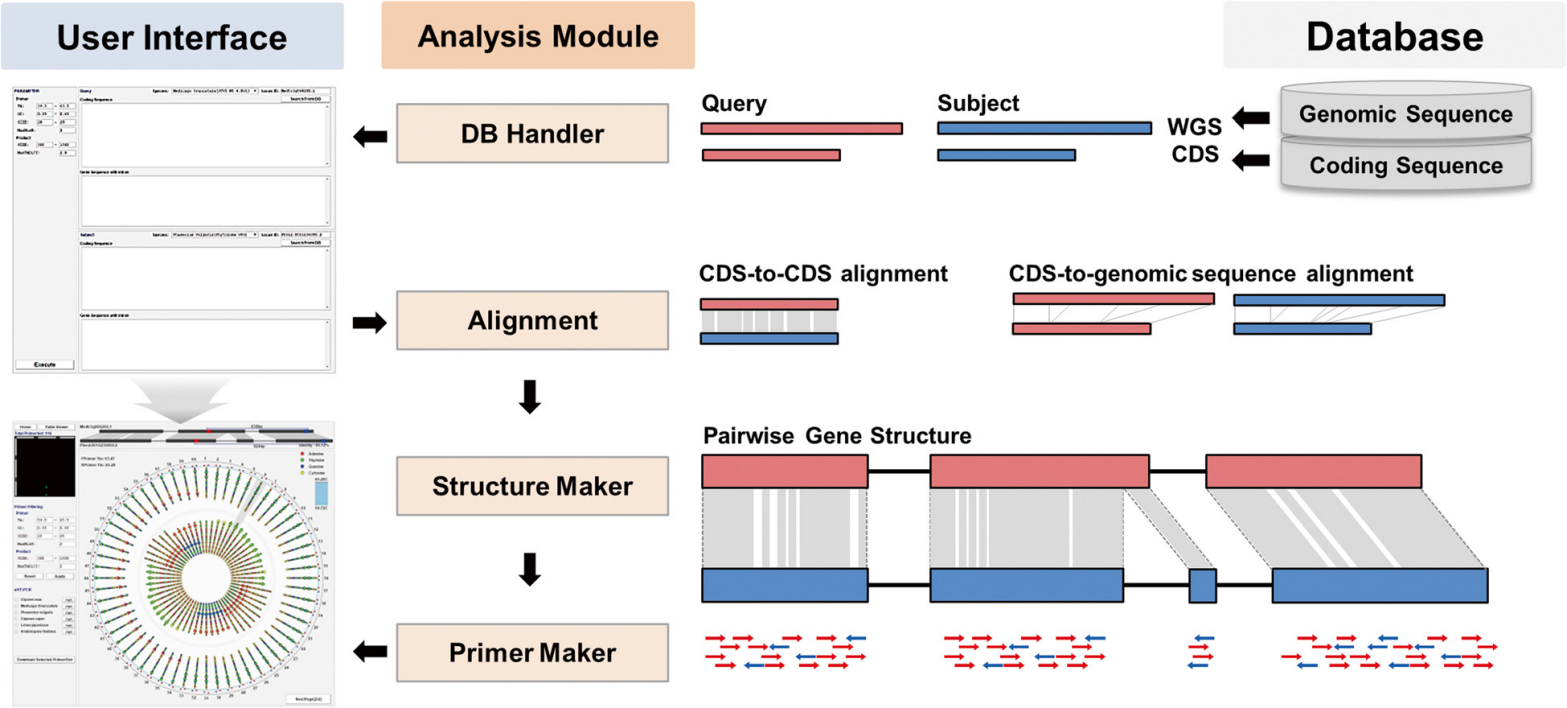
Organization and workflow of the CSGM Designer platform. The platform consists largely of database, analysis module and user interface. The panes on left upper and lower corners depict the windows for data input and circular viewer of results, respectively. Forward and reverse primers are denoted with red and blue arrows, respectively.

CSGM Designer consists of three parts: a user interface, an analysis module, and the underlying database (Figure 2). The user interface is structured around two main windows, one for data input with parameter control boxes, and one for the visualization of resulting primer pairs. The database includes information for CDS and genomic sequences of five substantially sequenced legume genomes (i.e., *M. truncatula*, *G. max*, *P. vulgaris*, *C. cajan* and *L. japonicus*) and Arabidopsis. The sequence data of target genes can be retrieved from the database using locus ID as a query, or by user provided candidate gene sequences that are compared to the database content. The user interface permits the user to adjust primer design parameters, and resulting primer pair candidates are displayed in the order of scores for individual primer pairs, either in a graphical “circular viewer” or in a “tabular viewer”.

CSGM Designer’s analysis module consists of two parts, “Primer Maker” and the eHT-PCR module. Primer Maker is a primer design module that incorporates two alignment algorithms. Splign [10] performs CDS-to-genomic sequence alignments with default options to locate exon-intron junctions. Simultaneously, the ClustalO program [11] conducts CDS-to-CDS alignment between gene pairs of full length. Splign and ClustalO outputs are used by Structure Maker to produce a pairwise gene structure, including the location of conserved and variable regions, which typically correspond to exons and introns. The output of Structure Maker provides the template for PCR primer design, which is carried out by Primer Maker.

Virtual amplification specificity of primer pairs created within the Primer Maker module is simulated by the eHT-PCR module. The BWA algorithm [12] was adapted for this purpose and serves a central role in the eHT-PCR module. Using BWA, numerous primer pair candidates are mapped against the reference genomes and evaluted for the specificity of cross-genome PCR amplification. By employing *in silico* PCR simulation prior to experimental practice, users can estimate how selected primer pairs will perform within and among the reference genomes, and by inference have utility in target genomes not included in the database.

### Features of CSGM Designer

**Figure 3:**
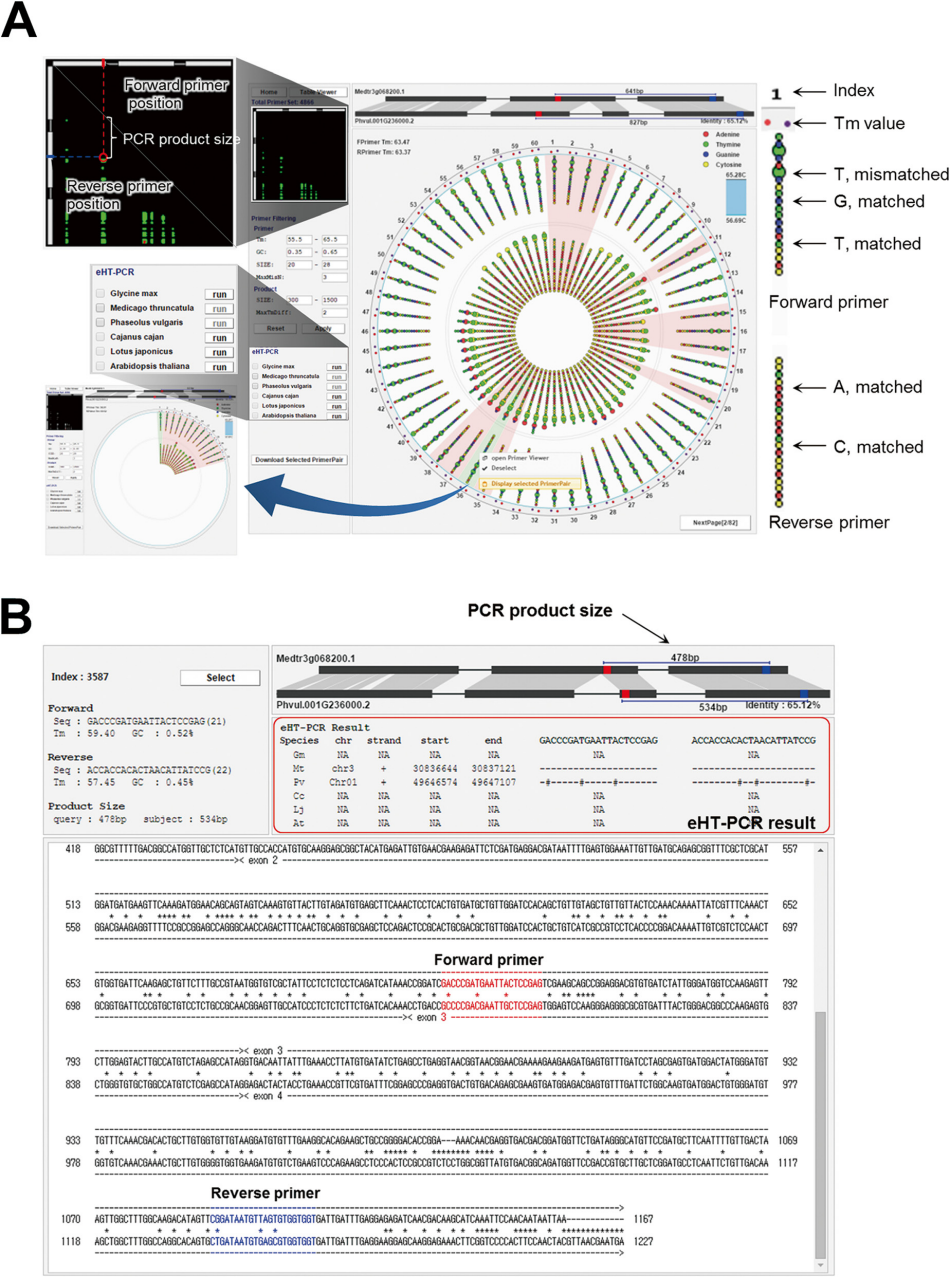
User interface for reviewing results of designed marker candidates. **A.** Circular viewer. The circular viewer presents a maximum of 60 primer pairs per page. Outer circle represents the forward primers, while inner circle the reverse primers. Each of four different nucleotides are denoted with different colors; red-A, green-T, blue-G and yellow-C. Mismatched nucleotides are highlighted using larger circles over matched ones. The pane on left upper corner display overall positional information for primers designed under selected parameters. Tm values for forward and reverse primers are depicted with red and blue circles, respectively, within minimum-to-maximum range, and accurate Tm values can be checked by selecting one of the primer pair candidates. Individual primer pairs of user’s interest can be selectively re-displayed using right button of the mouse over the circular viewer (the pane on left lower corner). **B.** Primer viewer. This viewer presents detailed information of selected primer pairs with aligned gene structure, corresponding genic sequence alignment, PCR product size and primer sequences. It also provides the results of eHT-PCR analyses.

**Figure 4:**
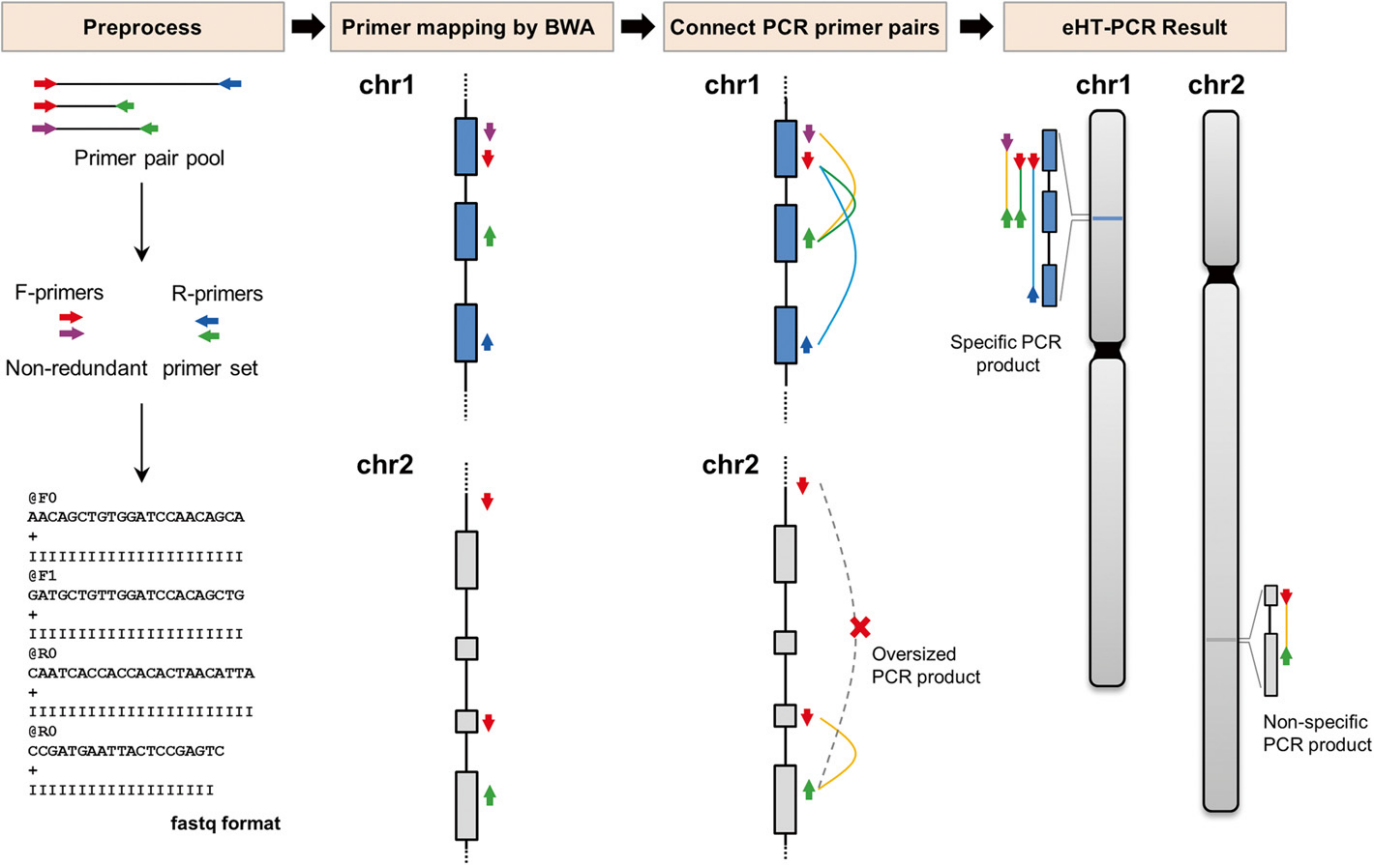
Concept of eHT-PCR and its workflow.

CSGM Designer incorporates a dynamic GUI that creates a “circular viewer” of primer candidates (Figure 3) and an “eHT-PCR” representation of virtual PCR outcomes (Figure 4), both of which are intended to enhance the user’s experience during cross-species marker development. The top-scoring 60 primer pairs are displayed in the first page of the circular viewer, providing the user with a graphical overview of otherwise complex query results. The viewer provides a range of information intended to guide the user towards the most suitable primer pairs, including primer melting point (Tm) values, oligonucleotide sequences, and mismatch distribution between primers and target sequences. Tm values permit the user to inspect differences between primer pair combinations. Colors are used to distinguish each of four different nucleotides, and mismatched positions are highlighted, providing a snapshot of the frequency and location of mismatched nucleotides (Figure 3A). Even for the best-conserved regions of candidate genes, mismatches are typical when sequences are compared among species. PCR is robust to such mismatch, assuming that mismatches have particular characteristics; here we limit the output to three mismatches, while the user can impose additional criteria. For example, mismatches in neighboring or consecutive sites and at the 3′-ends of primers, as well as AT-rich 3′ ends, can negatively affect PCR performance. The circular viewer facilitates such user-supplied criteria, enhancing success rates of PCR amplification.

CGSM Designer’s “overview pane”, on the upper left corner of the circular viewer, presents positions of all PCR primers within the context of target gene structure (Figure 3A). PCR product size can be visually estimated in the overview pane. The overview pane also provides a simple visual assessment of the relationship between primer positions and exon-intron distribution, revealing the number of introns included in virtual PCR products and whether their location of primer pairs is evenly distributed or clustered.

While the circular viewer provides a search-at-a-glance perspective, users can also investigate expanded details of chosen primer pairs in a “primer viewer” panel. In this viewer, users encounter further details of chosen primer pairs, including the location of primer hybridization and of mismatched bases in the context of sequence text. Genomic structure of aligned genes provides information on introns-exon structure, including nucleotide conservation/variation and PCR product sizes (Figure 3B).

PCR primer design can be further analyzed by the “search-within-result” function, to narrow primer candidates based on user provided criteria. Post-processing refinement allows the user to revisit parameters used for the first round of analysis, thereby increasing selectivity for primer candidates based on the user’s needs. Users can also directly select multiple primer pairs, with the selected primer pairs automatically incorporated into CSGM’s other viewer functions (Figure 3A).

Secondly, specificity of cross-species PCR amplification can be refined and guided by results from the eHT-PCR module. Reduced-specific PCR amplification can be difficult to overcome, especially in cases of DNA fragments derived from conserved gene families. The eHT-PCR analysis can be conducted using full genomic sequence information for any of the six species contained within the CSGM Designer database. Users can select all or a subset of species for analysis and the results can be seen in the primer viewer pane (Figure 3B). A specific virtual amplicon (i.e., single genomic locus) is represented with its corresponding information about genomic position of species selected for eHT-PCR, while results of non-specific PCR products (i.e., two or more genomic loci) are displayed by number of possible PCR amplifications.

eHT-PCR preprocessing pipeline is illustrated in Figure 4. The eHT-PCR simulation tool is performed in three steps: i) generation of non-redundant primer set by removing redundant primers from the initial pool of total primer candidates, ii) mapping the reduced primer set to selected genomes using the BWA program, and iii) predicting amplified genomic sites based on BWA mapping results (Figure 4). In conjunction with BWA-based genome-wide mapping of primer candidates, CSGM Designer can effectively reduce the number of possible forward/reverse primer combinations by removing redundancy in primer pools and false primer pairs (Figure 4). Subsequent combinations of forward and reverse primer pools are used to generate a list of virtual amplicons. Because eHT-PCR can be implemented for all six species in the database, users can rapidly determine which primer pairs are likely to be specific or non-specific PCR amplicons, and whether this prediction varies among species.

### PCR primer pair design using CSGM platform

CSGM Designer platform is intended to be intuitive. The step-by-step primer design can be processed according to the following procedures. A graphic-aided easy-to-use tutorial is also available on the CSGM homepage (http://tgil.donga.ac.kr/CSGMdesigner/tutorial.html).

(i) To design primer pairs, the CSGM Designer requires both genomic and coding sequences from at least two species. Users can retrieve the sequences of target gene using locus IDs from the database or the user can directly input candidate gene sequences for customized design using user-specified parameters.
(ii) By clicking “execute” button, CSGM Designer renders the pairwise gene structure, and subsequently the circular viewer allows visualization of designed primer pair candidates. Alignment results for chosen primer pairs are displayed along with detailed information about the primers and their virtual PCR products. Users can further select multiple primer candidates by clicking individual primer pairs in the circular viewer to arrive at a final decision.
(iii) To test and select primer candidates for specific PCR amplification at the genome scale, the user can invoke eHT-PCR within all or a subset of species. This *in silico* PCR is conducted in high throughput manner. Once done, primer pairs predicted to produce non-specific PCR amplicons can be removed from the candidate pool. Users can further reduce the number of primer pair candidates by applying more stringent PCR parameters.
(iv) Thereafter, users can view the full results for selected primer pairs, reconfirm the quality of individual or groups of primer pairs, and make a final decision based on amplification specificity, product size, the number of introns, Tm value and other factors. Users may download the selected primer pairs and related information by exporting the output as an Excell file.

### Experimental validation of the CSGM Designer

**Figure 5:**
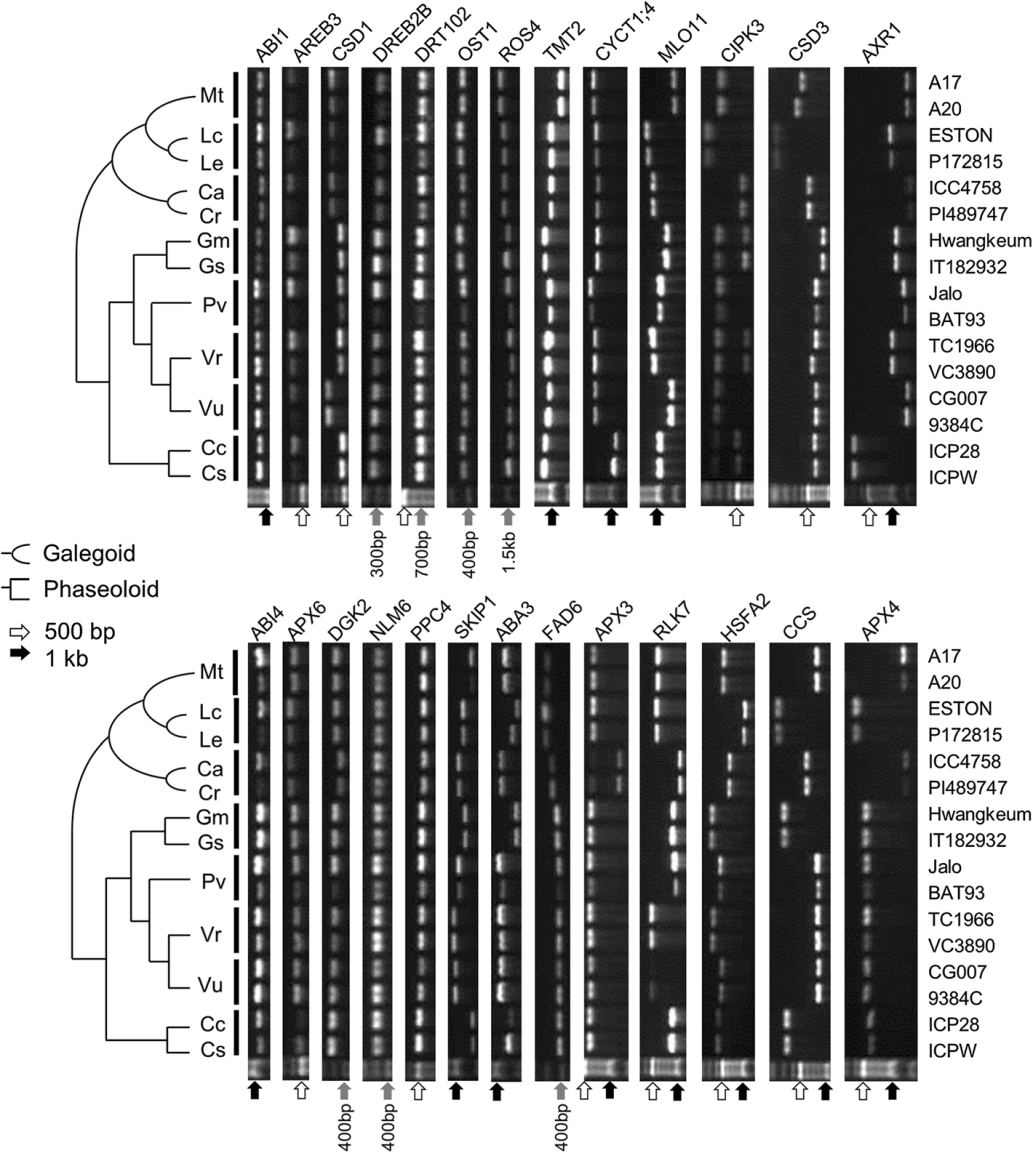
Cross-species PCR amplification of genic marker candidates. Twelve species employed for the experimental validation are organized according to their phylogenetic relationships on the left side. A total of sixteen genotypes were used and denoted on the right side. The abbreviations for each species denote their names as follows. Mt: *Medicago truncatula*, Lc: *Lens culinaris*, Le: *Lens Ervoides*, Ca: *Cicer arietinum*, Cr: *Cicer reticulatum*, Gm: *Glycine max*, Gs: *Glycine soja*, Pv: *Phaseolus vulgaris*, Vr: *Vigna radiata*, Vu: *Vigna unguiculata*, Cc: *Cajanus cajan*, Cs: *Cajanus scaraboides.*

**Figure 6:**
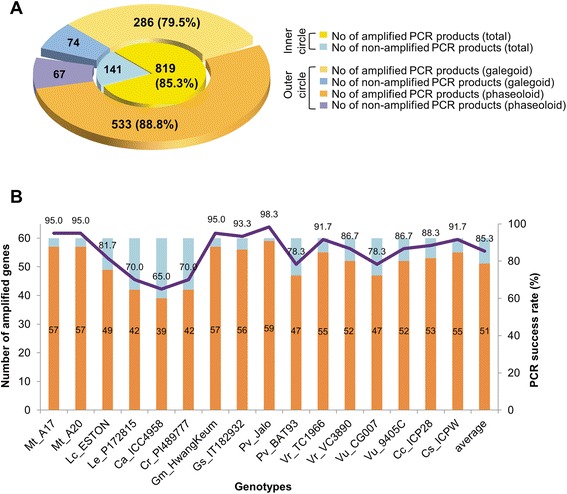
Summary of cross-species amplification of 60 selected abiotic stress-responsive genes. **A.** PCR success rates of overall cross-species and cross-clade amplifications. **B.** PCR success rates according to 16 genotypes. Pale brown bars and numbers indicate the number of genes amplified out of 60 selected genes. Solid line and numbers denote percent success rate of PCR amplifications. Abbreviated species names are the same as in Figure 5.

To test reliability of CSGM Designer output, a pilot scale experiment was performed. A total of 60 genes (Additional file 1: Table S1) were selected from a gene set associated with abiotic stress responses published in previous study [13]. A corresponding set of 60 PCR primer pairs, one for each target, was obtained using CSGM Designer. For this particular anlaysis, the genomes of *M. truncatula* (galegoid) and *P. vulgaris* (phaseoloid) were selected anchor species, with the expectation that resulting primers would have functionality for species in both clades. The *M. truncatula* A17 genome was used as the template sequence. The resulting 60 primer pairs were tested in PCR amplifications using 16 legume genotypes representing 8 genera and 12 species. The selected species are well distributed between the galegoid and phaseoloid clades and include both species contained within the database as well as species that lack substantive genomic resources. A total of 960 PCR reactions were performed and visualized by agarose gel electrophoresis, from which the success rate of PCR amplifications was calculated. Representative PCR amplification for 26 genes is shown in Figure 5, with success rates summarized in Figure 6. Of 960 PCR reactions, 819 PCR products were single bands, corresponding to 85.3% amplification. 25 genes (41.7%) were amplified across all 16 genotypes. PCR success rate for galegoid and phaseoloid species was 79.5% (286 amplicons/360 PCR reactions) and 88.8% (533 amplicons/600 PCR reactions), respectively (Figure 6A). These results demonstrate efficient cross-clade PCR amplification of conserved genes. Depending on species and genotypes, PCR success rates ranged from 65.0% (*C. arietinum* ICC4958) to 98.3% (*P. vulgaris* Jalo) (Figure 6B). Average success rate of *M. truncatula* and *P. vulgaris*, which were used as anchoring reference genomes, were 95.0% and 88.3%, respectively (Figure 6B).

Of 60 cross-species genic marker candidates, 9 genes (i.e., 144 PCR amplicons) were selected for the purpose of marker development. Of these, 130 sequences (i.e., 65 sets of parental lines) of high quality (GenBank accession numbers: KM047242-KM047371) were obtained and used to develop CAPS and/or SNP markers. Sequence comparisons of the 65 parental sets allowed us to detect nucleotide polymorphisms in 47 pairwise alignments (72.3%). Of these 47 SNP-bearing marker candidates, 37 parental sets (78.7%) were suitable for the CAPS marker development. Cleavage patterns of amplified PCR fragments were predicted and reconfirmed by restriction enzyme digestions, of which three genic CAPS markers are exemplified in Additional file 2: Figure S1. Entire marker information for SNP, cleavage patterns, restriction enzymes is available in Additional file 3: Table S2.

## Discussion

CSGM Designer is an easy-to-use platform that can automatically produce primer pairs, and functions efficiently as the basis for cross-species gene-based markers. Functionality and efficiency of the platform are enhanced by intuitive graphical interfaces, including a circular viewer that provides details of primer-target alignments (Figure 3) and an eHT-PCR module that represents the outcome of genome-wide *in silico* PCR amplification (Figure 4). Reference genome information from five legume species (Figure 2) is leveraged to create PCR primers that operate within the taxonomic space of the majority of legume crops. The platform’s reliability was validated by testing cross-clade PCR amplification and subsequent SNP detection and genetic marker development (Figures 5 and 6, Additional file 3: Table S2) for 12 legume species. The concept of highly conserved, cross-species intron-spanning genic markers is not new, and was first widely implemented by Choi et al. [3,14]. Importantly, the utility of these markers as phylogenetic tools and their ability to routinely identify orthologous genes even for uncharacterized species has been demonstrated across the entire legume family [15]. By analogy, the markers developed using CSGM should, in a high percentage of cases, also extend across the entire legume family. Moreover, similar approaches should have relevance for other plant families of agricultural importance, where well characterized and poorly characterized species co-occur.

Similar databases have been constructed for both legumes and grasses [16], however such analyses were implemented using incomplete information of genome and/or EST sequences. The recent completion of whole genome sequencing for several important model and crop legumes provides the basis for revisiting such approaches using genome-wide data. The advent of genome-wide data underlies a range of NGS-based studies in plants, including RNAseq-based transcriptome analyses [17,18] and resequencing-based GWAS (genome-wide association study) analyses [8,19]. Here, based on a multi-species comparison, we extend these benefits to species whose genomes have not been sequenced, but are related to the sequenced reference species. Genome structures of plant species are frequently complex, in particular due to segmental duplication and polyploidization [20,21]. CSGM designer would have greatest utility of single or low copy fractions of legume genomes, though CSGM also permits the identification and *in silico* PCR testing of candidates even for large gene families.

Reference-guided genome resequencing is routinely used to discover nucleotide variation at the genome scale, and given access to appropriate populations to associate traits of interest with polymorphisms. But the power of such approaches depends in large part on access to substantial genome sequence data. Alternative methods are required for crops where genome data is lacking. Towards this end, CSGM Designer provides the infrastructure for high throughput development of gene-associated molecular markers that can link poorly characterized target species with the increasingly well-characterized genomes of the reference legumes.

## Conclusions

In this study, we present the development and validation of CSGM designer, a new legume-focused bioinformatics platform for designing cross-species genic markers. Integration of reference legume genome information, interactive graphical content, and genome-wide *in silico* prediction of PCR amplification are key features of the platform. CSGM designer allow users, with minimal effort and high precision, to select marker candidates and test their efficacy *in silico* using genome-wide predictions of PCR amplification, as a prelude to actual experimentation. We expect that CSGM Designer have particular utility for poorly characterized legume species. In particular, connections to well characterized genomes through a network of highly conserved genes will enhance the potential for functional inference based on homology and synteny, and in doing so create a synergy for trait-associated molecular breeding.

## Methods

### Resources for the DNA sequence data

To construct WGS DB-associated marker designing platform, we used whole genomic information for five legume species (*Medicago truncatula, Glycine max, Phaseolus vulgaris, Lotus japonicas* and *Cajanus cajan*), whose genomes were recently fully sequenced and publically available. In addition to these five legumes, Arabidopsis was also included with the main intent of extracting functional annotations. Detailed genome information for six model and crop species is available in Table 1. Genome sequences for *G. max*, *P. vulgaris* and Arabidopsis were downloaded from Phytozome DB (http://www.phytozome.net). Genome data for *M. truncatula, Lotus japonicas* and *Cajanus cajan* was obtained from http://www.jcvi.org/medicago/, http://www.kazusa.or.jp/lotus/, and http://www.icrisat.org/gt-bt/iipg/Home.html, respectively. Genomic sequences of individual genes were extracted from the WGS data of each species provided by the general feature format (GFF) files. Coding sequences (CDS) were directly downloaded from corresponding databases and used to identify exon-intron junctions based on CDS-to-genomic sequence alignments.

**Table 1:** **Summary of genomic information on six species used for CSGM designer platform**

**Species name**	**Common name**	**Genome size (Mbp)**	**Chromosome No**	**Release version**	**Number of transcript**
*Arabidopsis thaliana*	Arabidopsis	157	n = 5	Phytozome v9.0	35,386
				Phytozome v10.0	35,386
*Glycine max*	Soybean	1,115	n = 20	Phytozome v9.0	73,320
				Phytozome v10.0	88,647
*Medicago truncatula*	Barrel medic	470	n = 8	Mt 4.0 v1	62,319
*Phaseolus vulgaris*	Common bean	625	n = 11	Phytozome v9.0	31,638
				Phytozome v10.0	31,638
*Lotus japonicus*	Birdsfoot trefoil	471	n = 6	Release 2.5	38,482
*Cajanus cajan*	Pigeon pea	833	n =11	Nov. 6, 2011	48,680

### Constitution of the CSGM Designer platform

The data processing pipeline of the CSGM Designer involves a combination of PHP and Javascript languages, while processed WGS and CDS data are organized within a MySQL relational database. Two programs, ClustalO (http://www.clustal.org/omega/) and Splign (http://www.ncbi.nlm.nih.gov/sutils/splign/splign.cgi), were employed to process two stepwise alignments. The ClustalO [11] was used to make CDS-to-CDS alignments and subsequent identification of the best-conserved regions within exons between two aligned coding sequences. The Splign algorithm [10] was used to align cDNA sequences with genomic DNA as a means to identify exon-intron junctions and exon-to-exon junctions within aligned CDSs. Structure Maker is a module that integrates the two types of alignment results and generates a corresponding pairwise gene structure. Based on the pairwise gene structure, Primer Maker was developed to extract gapless regions (i.e., continuous well conserved sequences) and to design paired primer candidates that avoid exon-intron junction sites. The Javascript was used to produce the graphic user interface (GUI) for the purpose of dynamic interactions between users and the GUI.

### Development of the eHT-PCR analysis module

We adapted the Burrows-Wheeler Aligner (BWA, http://bio-bwa.sourceforge.net/) [12] to develop an electronic high throughput PCR (eHT-PCR) analysis module. BWA was developed to map short sequence reads produced by the Next Generation Sequencing (NGS). Its ability to align short DNA sequences, makes the BWA program useful for electronic PCR in that i) PCR primers are short DNA sequence, ii) single primers can be evaluated at multiple loci within the genome, iii) mismatch rates can be flexibly controlled, facilitating cross-species primer design. In conjunction with BWA, the eHT-PCR data processing pipeline was constructed as follows. First, redundant primers were removed from a large number of PCR primer pairs produced by the CSGM Designer, resulting in non-redundant primer set. Secondly, the non-redundant primers were mapped on selected genomes using BWA with a maximum of five mismatches (parameters: −N -k 2 -i 30 -d 30 -n 5). Finally, the eHT-PCR graphic displays the number and positions of electronically simulated PCR products, including amplicon size among species.

### Plant materials and DNA isolation

A total of 16 legume genotypes, selected from and 12 species among 8 genera, were used for experimental validation of the CSGM Designer: *M. truncatula* cv. Jemalong A17 and A20, *Lens culinaris* ESTON, *Lens Ervoides* P172815, *Cicer arietinum* ICC4958, *Cicer reticulatum* PI489777, *Glycine max* cv Hwangkeum, *Glycine soja* IT182932, *P. vulgaris* Jalo and BAT93, *Vigna radiata* TC1966 and VC3890, *Vigna unguiculata* CG007 and 9405C, and *C. cajan* cv. ICP28 and *C. scaraboides* ICPW. Seed sterilization, scarification, germination and growth for *M. truncatula* were conducted according to publically available protocol (http://medicago.org/documents/Protocols/seedgerm.html). Other legume seeds were sterilized with 5% bleach for 3 minutes. After germination, seedlings were grown in incubators at 16 hr day period and 25°C. Young leaves were sampled for the DNA isolation. DNA was isolated using Exgene™ Plant SV kit (GeneAll, ROK) according to manufacturer’s instruction and concentrations of each DNA sample were equalized to 10 ng/ul for PCR reactions.

### PCR amplification & marker development

10 ul PCR reactions contained 1.0 unit of ExPrime Taq DNA polymerase (GenetBio, ROK), 1X reaction buffer, 0.25 mM of each dNTP, 2.5 pmole of each F/R-primer, 2.5 mM MgCl2 and 1 ng template DNA. PCR thermo-cycling was conducted with a 5 min initial denaturation step, followed by 35 cycles of 94°C for 30 sec, 55 ~ 57°C for 30 sec, and 72°C for 2 min, with a final extension for 10 min at 72°C. Amplified PCR products were purified using ExoSAP-IT PCR Product Cleanup Kit (Affymetrix, Santa Clara, CA) according to the manufacturer’s protocol. BigDye terminator reagents were used for sequencing reactions as described previously [14]. The resulting sequences were aligned, edited and scored for single nucleotide polymorphisms using the MEGA5 program (http://www.megasoftware.net/). SNPs discovered between mapping parents were used to design CAPS (cleaved amplified polymorphic sequence) markers. Polymorphic sites were revealed by digesting PCR products with the appropriate restriction enzymes and subsequent agarose gel electrophoresis.

## Additional files

Additional file 1: Table S1. List of genes and primer information used to validate CSGM Designer. Additional file 2: Figure S1. Examples of cross-species genic markers. Restriction enzymes used to reveal polymorphisms are denoted in the parenthesis. Information for all other markers developed for experimental validation is available in Addional file 3: Table S2. Additional file 3: Table S2. Information of cross-species genic markers.

## Abbreviations

CSGM: Cross-species genic marker
eHT-PCR: Electronic high throughput PCR
GUI: Graphic user interface
BWA: Burrows-Wheeler aligner
CDS: Coding sequence
GFF: General feature format
WGS: Whole genome sequence
GBS: Genotyping-by-sequencing
QTL: Quantitative trait loci
GWAS: Genome-wide association study
