# Supplementary figures and images for: CSGM Designer: a platform for designing cross-species intron-spanning genic markers linked with genome information of legumes
Source: Plant Methods. 2015 Apr 18;11:30. doi: 10.1186/s13007-015-0074-6 (PMC4407554; doi:10.1186/s13007-015-0074-6)

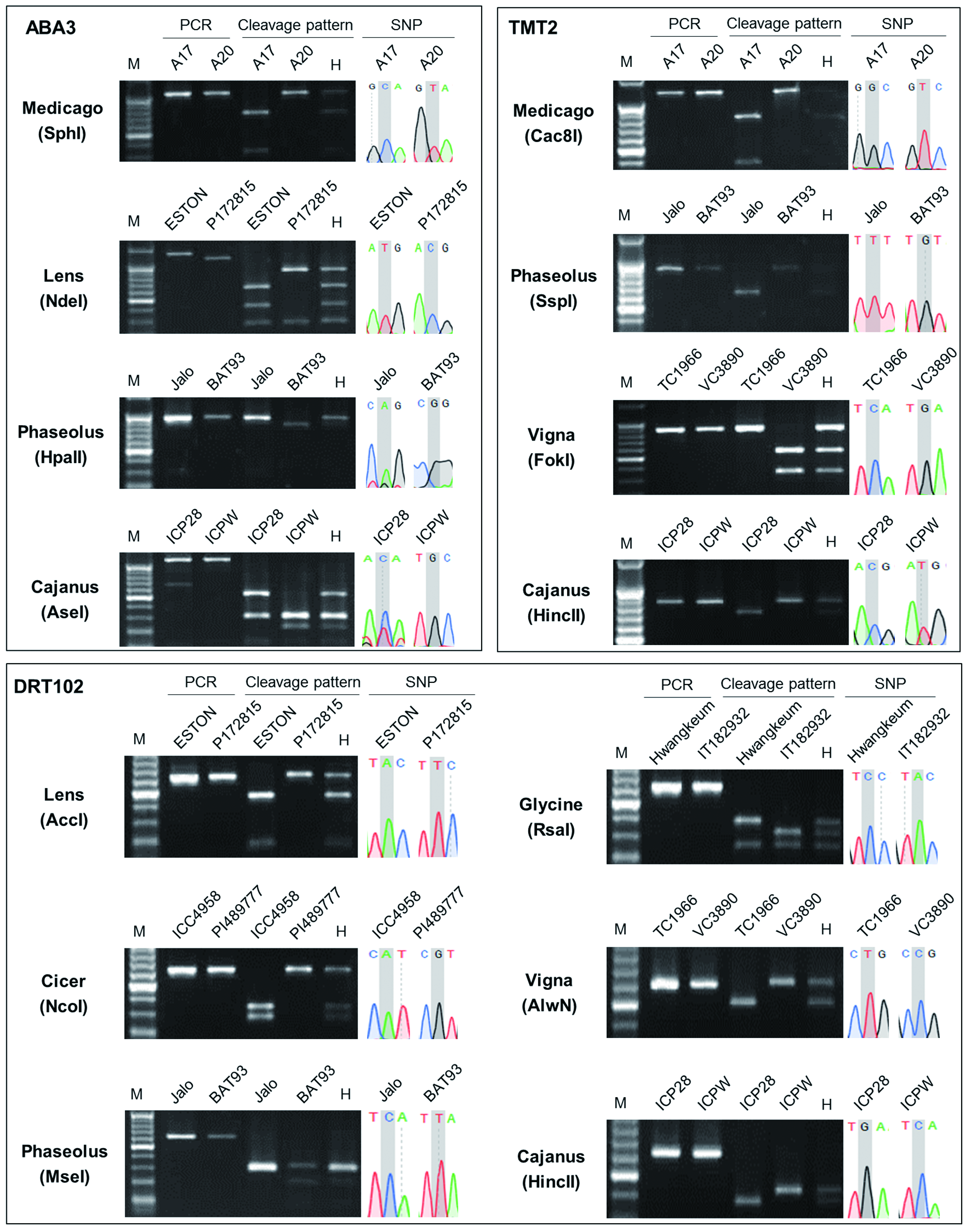

Supplement: Additional file 2: Figure S1. — Examples of cross-species genic markers. Restriction enzymes used to reveal polymorphisms are denoted in the parenthesis. Information for all other markers developed for experimental validation is available in Addional file 3: Table S2. [file 13007_2015_74_MOESM2_ESM.tiff]
